# Latent physiological factors of complex human diseases revealed by independent component analysis of clinarrays

**DOI:** 10.1186/1471-2105-11-S9-S4

**Published:** 2010-10-28

**Authors:** David P Chen, Joel T Dudley, Atul J Butte

**Affiliations:** 1 Program in Biomedical Informatics, Stanford University School of Medicine, Stanford, CA 94305, USA; 2 Departments of Pediatrics and Cancer Biology, Stanford University, Stanford, CA 94305, USA; 3 Lucile Packard Children's Hospital, 725 Welch Road, Palo Alto, CA 94304, USA

## Abstract

**Background:**

Diagnosis and treatment of patients in the clinical setting is often driven by known symptomatic factors that distinguish one particular condition from another. Treatment based on noticeable symptoms, however, is limited to the types of clinical biomarkers collected, and is prone to overlooking dysfunctions in physiological factors not easily evident to medical practitioners. We used a vector-based representation of patient clinical biomarkers, or clinarrays, to search for latent physiological factors that underlie human diseases directly from clinical laboratory data. Knowledge of these factors could be used to improve assessment of disease severity and help to refine strategies for diagnosis and monitoring disease progression.

**Results:**

Applying Independent Component Analysis on clinarrays built from patient laboratory measurements revealed both known and novel concomitant physiological factors for asthma, types 1 and 2 diabetes, cystic fibrosis, and Duchenne muscular dystrophy. Serum sodium was found to be the most significant factor for both type 1 and type 2 diabetes, and was also significant in asthma. TSH3, a measure of thyroid function, and blood urea nitrogen, indicative of kidney function, were factors unique to type 1 diabetes respective to type 2 diabetes. Platelet count was significant across all the diseases analyzed.

**Conclusions:**

The results demonstrate that large-scale analyses of clinical biomarkers using unsupervised methods can offer novel insights into the pathophysiological basis of human disease, and suggest novel clinical utility of established laboratory measurements.

## Background

Factorial methods have been successfully applied to diverse forms of multidimensional biological and clinical data to uncover both biological and clinical phenomena. Principal Component Analysis (PCA) and Independent Component Analysis (ICA) have been applied to gene expression microarrays to uncover latent factors in the expression data related to cellular processes, transcriptional control programs, and disease subtypes [1-4]. ICA has also been successfully applied to fMRI brain imaging data to uncover nondeterministic signals of interest [5].

ICA is a powerful extension of PCA originally developed as a solution to signal separation problems. PCA can only impose statistical independence of components up to the second order; therefore it can only identify directions that are uncorrelated and orthogonal to each other. ICA is capable of exploiting higher-order statistics to relax the orthogonality assumption and identify components that are mutually statistically independent from each other, which is a stronger condition than lack of correlation [6]. Given that ICA aims to find components that are non-Gaussian, it has the desirable side effect of ignoring variance resulting from noise in the data. It is likely that this higher-order model is more reflective of biological phenomenon, and thus offers an explanation for the many successful applications of ICA in the biomedical domain.

In previous studies, we demonstrated the utility for patient clinical biomarker vectors, which we termed clinarrays, in distinguishing between different severities of disease [7]; in integrative analysis with gene expression measurements to elucidate genes related to maturation and aging [8]; and to build a model of pediatric aging [9]. Clinarrays are a vector representation of a person’s physiological biomarker state across all visits at a hospital. Each value in this vector represents a summary statistic of a particular biomarker across a period of time. Aggregations of clinarrays enable the use of methods developed for microarray analysis to gain new insights into the clinical characteristics of disease-affected patients.

**Figure 1:**
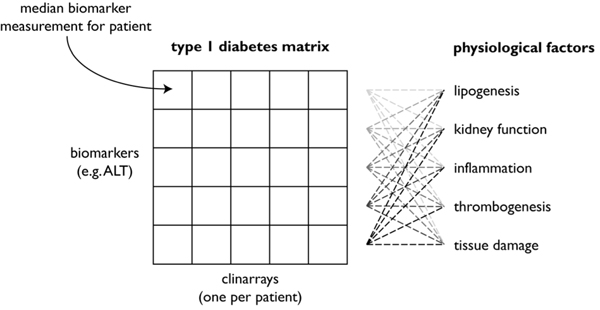
**Visual schematic of the model of ICA model of disease pathophysiology** ICA identifies mutually statistically independent latent physiological factors in the biomarker data. Each observed patient clinarray is modeled as a linear combination of the underlying factors whose coefficients are stored in the mixing matrix.

In this study we seek to apply ICA to clinarrays to identify latent physiological modes underlying chronic disorders. Figure 1 demonstrates the ICA model of in the context of clinarrays. We hypothesize that patients present diseases as unique manifest combinations of latent physiological factors (e.g. varying degrees of systemic inflammation and hyperinsulinism), and that a subset of highly discriminating latent factors can be used to differentiate patients diagnosed with the same disease.

We demonstrate that ICA can be applied to clinarrays of patient lab measurements to uncover physiological factors known to be concomitant in common chronic diseases. Furthermore, we identify a novel latent physiological factor for cystic fibrosis that serves as a promising lead for further investigation into diagnostic or prognostic biomarkers for CF.

## Results

### Creation of disease-specific clinarrays

Patients diagnosed with one or more of 50 International Classification of Disease (ICD-9-CM) codes representing five chronic conditions were retrieved from Lucile Packard Children’s Hospital. In total, this consisted of 4,085 patients (Additional file 1). Five disease-specific matrices were created with rows representing biomarkers and columns representing individual patient clinarrays. The value of each cell in a disease-specific matrix represents the median biomarker value for a single individual across all hospital visits. Patients were removed if they did not have at least 10 different biomarkers measured. Biomarkers were removed if they were not measured in at least half of the remaining patients. After removing patients and biomarkers with too few measurements, we were left with between 56 and 1,899 patients and between 21 and 58 biomarkers across the five diseases (Table 1).

**Table 1:** Number of patients and biomarkers remaining after pruning

	Patient count	Biomarker count
Asthma	1,899	29
Type 1 diabetes	343	21
Type 2 diabetes	413	31
Duchenne muscular dystrophy	56	58
Cystic fibrosis	335	44

The number of patients remaining in the data set after applying data filters is shown (see Methods). Biomarker count refers to the number of distinct types of laboratory measurements available for each patient in the disease set after filtering.

### Independent component analysis of clinarrays

Each clinarray was subjected to iterative ICA analysis and the significant biomarkers for each disease were derived using the approach detailed in the methods (Table 2). We manually assigned each biomarker a physiological process descriptor that it was representative of its clinical utility as detailed by a clinical laboratory reference[10]. For example, an increase in ALT or AST is generally associated with tissue injury. Serum sodium was found to be the most significant factor for both type 1 and type 2 diabetes, and was also significant in asthma. TSH3, a measure of thyroid function, and blood urea nitrogen, indicative of kidney function, were factors unique to type 1 diabetes respective to type 2 diabetes. Lactate dehydrogenase, and indicator of tissue injury, and triglycerides were found to be significant only in Duchenne muscular dystrophy; whereas total IgE, a marker of antibody response, and alkaline phosphatase levels were significant only in cystic fibrosis. Only platelet count was significant across all the diseases analyzed.

**Table 2:** Significant biomarkers after ICA analysis

	Significant Biomarkers	Physiological Processes
**Asthma**	Platelet Count	Thrombogenesis
		Serum Sodium	Serum sodium
		ALT	Tissue injury
		Neutrophil Percent	Acute inflammation

**Type 1 Diabetes**	Serum Sodium	Serum sodium
		Blood Urea Nitrogen	Kidney function
		Platelet Count	Thrombogenesis
		TSH3	Thyroid function

**Type 2 Diabetes**	Serum Sodium	Serum sodium
		ALT	Tissue injury
		Platelet Count	Thrombogenesis

**Duchenne Muscular Dystrophy**	Lactate Dehydrogenase	Tissue injury
		Triglycerides	Lipogenesis
		Platelet Count	Thrombogenesis
		AST	Tissue injury

**Cystic Fibrosis**	Total IgE	IgE antibody response
		Alkaline Phosphatase	Dephosphorylation
		Platelet Count	Thrombogenesis

Significant biomarkers for each disease are shown. Each significant biomarker was identified as a significant and statistically independent physiological factor from the patient laboratory data by ICA analysis (see Methods). Each significant biomarker was matched to a broader physiological process using a standard reference for clinical laboratory chemistry.

## Discussion

We applied ICA analysis to biomarker clinarrays in an effort to elucidate latent physiological factors concomitant in five chronic disorders. Overall we find associations that are consistent with known physiological factors of the diseases studied, showing that the factors extracted by the ICA algorithm are reflective of actual physiological processes underlying each disease. It is interesting to note that platelet count, which we use as a marker for thrombogenesis, appears to be the only factor shared across all of the diseases studied. Recent studies have revealed physiological roles for platelets that extend well beyond thrombogenesis, including roles in innate immunity, microbial defense, and angiogenesis[11]. Our findings suggest that platelet activation may be a salient pathophysiological factor for a broad range of chronic disorders.

We investigated biomarkers of patients with Cystic Fibrosis (CF), a hereditary multi-organ inflammatory disorder caused by mutations in the *CFTR* gene. The results identify three significant physiological factors latent in the disease pathology: IgE antibody response (Total IgE), dephosphorylation (alkaline phosphatase) and thrombogenesis (platelet count). Platelet activation has been implicated as a major factor in the pathogenesis and progression of CF. Falco and colleagues suggest that platelets participate in the pathogenesis of CF by increasing levels of soluble CD40 ligand, whose secretion has been linked to coagulation activity [12]. These findings elucidate a putative role for platelet activation in disease progression, and inform the course of treatment for patients exhibiting high degrees of platelet activation[13].

A clear pathophysiological explanation for alkaline phosphatase (ALKP) is not immediately evident. Differential levels of ALKP in CF may be attributed to bone disease[14] or biliary obstruction[15]. The interpretation is confounded by the fact that the data was derived from pediatric patients who may have greater variance of ALKP, due to differential rates of bone growth. However, no other disease resulted in significance for ALKP, even though the range of ages was similar for the other diseases. ALKP remains a potentially novel finding.

One of the more interesting findings from a clinical perspective is the identification of an Immunoglobulin E (IgE) component in CF. IgE plays an important role in allergic sensitivity and response [16]. IgE levels are known to be elevated in a subset of CF patients due to allergic bronchopulmonary aspergillosis (ABPA), an allergic reaction to a secondary fungal infection of an aspergillius species[17]. The prevalence ABPA in pediatric CF patients is estimated to be anywhere from 2% to 15%[18]. Despite the fact that detection and treatment of fungus has been known to improve prognosis, it is not common practice to screen for fungus. Testing for ABPA in CF patients is not usually conducted until the patient fails to respond to antibiotic therapy for an extended period. Our findings suggest that ABPA may be more of a significant factor among patients seeking treatment for CF symptom which suggests a more routine screening for IgE antibodies to mitigate prolonged lung damage and improve patient prognosis.

Type 1 and type 2 diabetes, though similar in regards to causing an abnormal increase in blood sugar, have etiological roots in distinctly different pathophysiological mechanisms. Both types of diabetes are known to cause hyperglycemia, which decreases serum sodium concentrations due to water exiting the intracellular to the extracellular space [19]. Hyperglycemia is known to be associated with decreased measured serum sodium [20]. Therefore the identification of serum sodium as a significant factor for both forms of diabetes by our analysis is concordant with expectations from established pathophysiology. Sterner and colleagues showed that an increased platelet count is associated with type 1 diabetes both with and before renal impairment [21]. Jesri and colleagues showed that platelet counts significantly increased as patients accrued more risk factors for metabolic syndrome [22], a precursor for type 2 diabetes. Therefore while platelet count was significant in all diseases, there is an established basis for its association with both types of diabetes analyzed.

Thyroid stimulating hormone (TSH) was found to be significant in distinguishing patients with type 1 diabetes, while it was conspicuously absent from our analysis of type 2 diabetes. Thyroid autoimmunity affects approximately 20% of patients diagnosed with type 1 diabetes [23]. For patients with thyroid autoimmunity TSH levels were found to be significantly higher. Moreover, patients with two thyroid antibodies had an even higher TSH level [24]. As expected, we did not find this biomarker in type 2 diabetes. Similarly, we recovered a serum alanine aminotransferase (ALT) association with type 2 diabetes. It has been previously shown that elevated ALT was associated with insulin resistance, the main component of the pathogenesis of type 2 diabetes [25]. We also note that certain biomarkers with known associations to type 1 diabetes and type 2 diabetes, such as hemoglobin A1c, are not returned in our analysis. This may be due to the inability of these biomarkers to differentiate between patient subsets within these particular diseases, or a lack of statistical independence in the associated data.

We also examined Duchenne muscular dystrophy (DMD), a disease whose pathogenesis stems from a mutation in the dystrophin gene. It was previously shown that patients with DMD may have elevated levels of AST, which is corroborated by our analysis [26]. These high levels of AST have been attributed to muscle breakdown rather than liver pathology, which is traditionally associated with AST levels. As more muscle begins to break down in the progression of DMD, we would expect to see an increase in levels markers for tissue damage, such as AST. Therefore we suggest that AST may serve as a marker for disease progression in DMD. It also has been shown that lactate dehydrogenase (LDH), also identified by our analysis, is increased in patients affected with DMD [27].

A relationship whose basis is less evident is the implication of triglyceride levels as a factor in DMD. Young and colleagues have shown that mice with hereditary muscular dystrophy have significantly increased amounts of triglyceride in skeletal muscle biopsies [28]. There has been a conspicuous absence of examining triglyceride levels in the blood for DMD patients, which is captured in clinical lipid profiles. A recent study by Wren and colleagues showed that muscle adiposity values are accurate in determining disease severity of DMD patients [29]. Our analysis suggests that triglyceride levels in blood could also serve as a novel biomarker that indicates the severity of DMD.

We acknowledge some limitations in our approach. Foremost, the range of lab measurements found in the patient data limited the identification of significant biomarkers for each disease. These biomarkers were also manually mapped to general physiological processes and thus are limited to our current understanding of physiology. Biomarkers may be involved in multiple physiological processes and may be part of processes we do not capture. We also acknowledge that clinarrays do not take into consideration the temporal aspect of disease progression. However, as with microarrays, the temporal aspect of gene expression within a given tissue is also often ignored unless specifically part of the protocol. ICA-based clinarray analysis could be extended to incorporate a temporal component in future work. We also note that while we currently do not use the longitudinal data available at a patient-specific level in electronic medical records, this type of data could be incorporated in future work to potentially increase the power of our analysis. In several cases, lab measurements could not be included in the clinarray for a disease as the underlying patient data was too sparse. Thus, the set of laboratory measurements was not the same for each clinarray. In addition, the use of ICA is only appropriate when the assumption of combinatorial linearity of components is satisfied. Further testing is needed to determine if this assumption holds generally for clinical biomarker data.

In future work we hope to expand our data set to include biomarkers for additional diseases and formulate clinical validation of the results as well as integrate longitudinal and temporal information to produce more extensive models of disease pathophysiology.

## Conclusions

We have developed a novel approach that incorporates independent component analysis of patient laboratory biomarkers represented as vectors in a clinarray. This approach seeks to separate out statistically independent signals of disease pathophysiology as measured by clinical laboratory tests on patient populations. We applied this approach to a data set characterizing five distinct disease conditions in a pediatric patient population. The results of this analysis yielded both unique and common physiological factors associated with the diseases analyzed. Several of these associations can be explained by known disease pathophysiology, whereas some of the associations are novel, and therefore suggest novel disease biology as well as novel uses for established laboratory biomarkers in clinical management of these diseases. This work provides a novel basis for unsupervised, data-driven analysis of disease pathophysiology from aggregate clinical laboratory measurements of patient populations.

## Methods

### Building clinarrays from patient lab tests

Quantitative clinical biomarker data, consisting of 893,956 measurements across 972 distinct biomarkers, obtained at the Lucile Packard Children’s Hospital (LPCH), were collected in a de-identified manner from the Stanford Translational Research Database Environment (STRIDE; http://stride.stanford.edu). This data represented 4085 patients diagnosed with one or more of five chronic diseases: asthma, type 1 diabetes, type 2 diabetes, cystic fibrosis, and Duchenne muscular dystrophy. The Institutional Review Board of the Stanford University School of Medicine approved the use of de-identified clinical data in this manner.

Clinarrays were generated from patients diagnosed with one or more of the diseases previously mentioned. The median value for each biomarker across all measurements of that biomarker for an individual patient was calculated. The median values for all biomarkers were aggregated to create the clinarray. Due to the paucity of data, thresholds were implemented to exclude patients and biomarkers that were poorly represented. Patients with less than 10 distinct biomarkers were excluded. Biomarkers without measurements for at least fifty percent of the remaining patients were also excluded.

### Independent component analysis

For our analysis we consider a clinarray data matrix **X** where the columns correspond to individual patient clinarrays, one clinarray per patient, and whose rows correspond to the variable biomarker measurements for each patient. Missing values were imputed using the K nearest neighbor imputation (KNNImpute) algorithm[30] with K = 10. The biomarker values were centered to a mean of zero and scaled to unit variance. The ICA model considers **X** as the matrix product of statistically independent components or signals **S**, and a mixing matrix **A**.

X = AS

Thus the observed physiological measures in the clinarray **X** are considered to be a linear combination of statistically independent latent physiological modes in various combinations as defined by the unknown mixing matrix **A**. Thus the goal of ICA is to estimate an optimal un-mixing matrix **W** that attempts to satisfy the equation:

Y = WX = WAS

Where **Y** is an approximation of **S** and if **W**=**A^-1^** then **Y** is a perfect reconstruction of **S**.

In this analysis we employed the FastICA algorithm proposed by Hyvärinen[31]. FastICA employs a contrast function that uses approximations of neg-entropy to identify non-Gaussian components. We determined the number of components to extract using the screen test method, which is used in exploratory factor analysis to estimate the putative number of latent principal factors.

### Extracting physiological factors

The results produced by the FastICA algorithm are influenced by random initializations from which the objective function of minimizing statistical independence of components is optimized. Thus the algorithm is known to find local minima depending on the initial conditions, and successive runs of the algorithm will produce slightly different results. In addition, ICA algorithms do not extract the components in order. Therefore we designed an iterative ICA analysis in which the FastICA algorithm was applied to the clinarray data over 500 iterations.

We investigated two different techniques for extracting significant laboratory biomarkers from the ICA iterations. First we examined the distribution of the absolute signal loading scores across all components and iterations. We then identified significant lab tests by identifying, for each disease, lab tests whose mean absolute component loading score was in the top 1% of the distribution. In a second approach, we counted number of times each lab was found to have the highest absolute loading score within a component across all iterations, and selected lab tests found to load highest on a component in all iterations. Both approaches yielded exactly the same results.

## Competing interests

The authors declare no competing interests.

## Authors' contributions

DPC and JTD conceived of the study, performed the analysis, and wrote the paper. AJB participated in the study design and wrote the paper.

## Supplementary Material

Additional file 1 **
				Additional file 1 – Characteristics of patient data extracted from clinical records for analysis** For each disease the ICD9 codes taken to represent the disease along with the count of patients identified using these codes is shown. Click here for file
