# Supplementary material for: Latent physiological factors of complex human diseases revealed by independent component analysis of clinarrays
Source: BMC Bioinformatics. 2010 Oct 28;11(Suppl 9):S4. doi: 10.1186/1471-2105-11-S9-S4 (PMC2967745; doi:10.1186/1471-2105-11-S9-S4)
Supplement: Additional file 1 — – Characteristics of patient data extracted from clinical records for analysis For each disease the ICD9 codes taken to represent the disease along with the count of patients identified using these codes is shown. [file 1471-2105-11-S9-S4-S1.doc]

| **Disease** | **ICD-9-CM** | **# Patients** |
| --- | --- | --- |
| Asthma | 493.00, 493.01, 493.02, 493.10, 493.12, 493.20, 493.21, 493.22, 493.81, 493.82, 493.90, 493.91, 493.92 | 2,668 |
| Cystic Fibrosis | 277.0, 277.00, 277.01, 277.02, 277.03, 277.09 | 379 |
| Duchenne Muscular Dystrophy | 359.1 | 63 |
| Type 1 Diabetes | 250.01, 250.03, 250.11, 250.13, 250.21, 250.31, 250.33, 250.41, 250.43, 250.51, 250.61, 250.63, 250.71, 250.81, 250.83,  250.91, 250.93 | 871 |
| Type 2 Diabetes | 250.00, 250.02, 250.10, 250.12, 250.20, 250.22, 250.40, 250.50, 250.60, 250.80, 250.82, 250.90, 250.92 | 673 |
